# A Method for Generating New Datasets Based on Copy Number for Cancer Analysis

**DOI:** 10.1155/2015/467514

**Published:** 2015-04-08

**Authors:** Shinuk Kim, Mark Kon, Hyunsik Kang

**Affiliations:** ^1^College of Liberal Arts, Sangmyung University, Cheonan, Chungnam 330-720, Republic of Korea; ^2^Department of Mathematics and Statistics, Boston University, Boston, MA 02215, USA; ^3^College of Sport Science, Sungkyunkwan University, Suwon 440-746, Republic of Korea

## Abstract

New data sources for the analysis of cancer data are rapidly supplementing the large number of gene-expression markers used for current methods of analysis. Significant among these new sources are copy number variation (CNV) datasets, which typically enumerate several hundred thousand CNVs distributed throughout the genome. Several useful algorithms allow systems-level analyses of such datasets. However, these rich data sources have not yet been analyzed as deeply as gene-expression data. To address this issue, the extensive toolsets used for analyzing expression data in cancerous and noncancerous tissue (e.g., gene set enrichment analysis and phenotype prediction) could be redirected to extract a great deal of predictive information from CNV data, in particular those derived from cancers. Here we present a software package capable of preprocessing standard Agilent copy number datasets into a form to which essentially all expression analysis tools can be applied. We illustrate the use of this toolset in predicting the survival time of patients with ovarian cancer or glioblastoma multiforme and also provide an analysis of gene- and pathway-level deletions in these two types of cancer.

## 1. Introduction

Copy number variations (CNVs) are promising DNA-level biomarkers of cancer subtype. CNVs can influence the phenotypes of cancer by disrupting (i.e., removing) or duplicating (i.e., adding) copies of a gene [1]. Although CNV studies have developed considerably over time [2–5], little is known about how CNVs affect cancer pathogenesis.

Previous studies describe several software packages useful for analysis of CNVs [6–8], primarily focused on identifying significant copy number alteration (CNA). One software package that is widely used for this purpose is CNVtools (http://cnv-tools.sourceforge.net/) [8], which deals with large CNA datasets. Other successful algorithms include Genomic Identification of Significant Targets in Cancer (GISTIC) [9] and its derivative JISTIC [10]. These algorithms identify regions with aberrant copy number using statistical calculations and then confirm related genes by matching chromosome regions. We consider CNV analysis from a different perspective: first, we reprocess the gene region, which we call the pseudogene, and then generate a new type of data, copy number alteration in array form (CNAR), at the gene level. We present more details regarding our approach and its applications in Methods and Results.

In the past decade, a number of useful and well-organized toolsets were developed for analyzing cancer phenotypes or genotypes. However, most of these software packages are commonly used to analyze gene-expression datasets. For example, unsupervised clustering methods and supervised classification methods are applied to machine learning algorithms in order to classify cancer phenotype, survival time, cancer metastasis, and so forth. Such algorithms create distinctions based on tissue RNA signatures for their predictive and classification tasks. Certain tissue samples contain macroscopic DNA variations which extend RNA variations based on CNVs, especially in cancer.

Particularly in cancer tissue, distinctions, classifications, and predictions based on such DNA variations may be useful. Indeed, DNA variation in a tumor changes more slowly than RNA variation and thus may be considered less noisy. Therefore, it is worthwhile to consider DNA-based algorithms.

If DNA copy number datasets (e.g., Agilent datasets) could be reprocessed into formats that parallel RNA expression signatures obtained from microarrays, it would be possible to construct DNA-based algorithms that parallel existing algorithms based on RNA. To the extent that a primary contributor to the expression level of a gene in cancerous tissue is the corresponding gene copy number, this type of analysis, using DNA copy number microarrays, could be considered as a proxy for RNA microarrays. Given that RNA signatures are more time-dependent than cancer-cell DNA signatures, the latter provide a more stable set of biomarkers for use in prediction of survival time, chemotherapy response, and other outcomes.

## 2. Materials and Methods

Here we present an algorithm which reprocesses Agilent DNA copy number signatures into a format that parallels the microarray signatures used in standard software packages for prediction and classification based on microarrays. Because copy number datasets are provided with probe IDs, we first need to download gene information to convert Agilent probe positions to gene regions. The following describes the steps for generation of CNARs corresponding to known genes.

**Step 1 (obtain datasets).**

We downloaded 216 ovarian cancer (OV) and 215 glioblastoma multiforme (GBM) CNA datasets, produced by Harvard Medical School using the Agilent Human Genome Comparative Genomic Hybridization Microarray 244A platform (HG-CGH-244A), updated in 2012 from the TCGA data portal. The portal also provided probe IDs with probe positions on the chromosome covered by 60 base pairs (bp). We also selected two classes of survival datasets for classification. From the GBM datasets, we extracted 23 samples from patients whose survival time was less than 100 days (short class) and 23 samples from patients with survival time greater than 1500 days (long class). From the OV datasets, we extracted 18 samples from patients whose survival time was less than 1 year (short class) and 18 samples from patients whose survival time was greater than 5 years (long class). The survival times distinguishing the long and short classes differed between GBM and OV because GBM is a more fatal disease.

**Step 2 (obtain gene and probe information).**

We downloaded Agilent genome region from BioMart (http://www.biomart.org/biomart/martview), which provides chromosome location for each HGNC_ID gene according to the HUGO gene nomenclature committee (http://www.genenames.org/) [1]. Out of the 22,734 genes initially downloaded from BioMart, the number of unique pseudogenes was 21,856. The HG-CGH-244A platform contains 227,612 probe IDs.

**Step 3 (extend the gene regions).**

We extended each gene region by including 2000 bp for the promoter region and 100,000 bp on each side, the so-called pseudogene. Each chromosome includes approximately 20 million base pairs, and the number of genes in each chromosome is about 1000. Therefore, an expansion of 100,000 bp on each side is reasonable. In addition, the region involved in CNV is commonly much larger than the size of a gene. We note that the range of CNV is commonly 1 kb or larger [12]. The number of probe IDs in the extended region is variable. The average number of probes for each gene is 37 and each probe includes 60 bp. Therefore an average pseudogene covers additional 2220 bp, but not successively connected positions between probes.

**Step 4 (make the base matrix).**

First, we generated a sparse matrix called the base matrix, with dimensions 21,856 (genes) by 227,612 (probe IDs). Each component *C*_*ij*_ of the base matrix reports the existence of a probe ID, *i*th, corresponding to a pseudogene *j*th by matching chromosome regions obtained from Steps 2 and 3. The component could be 1 or 0 for existence of probe a or the average of number of probes included in a corresponding gene presenting below b. *k* is the total number of the probe IDs in pseudogene *j*:

$$\begin{array}{cc} \left(\text{a}\right) & C_{ij}=\left\{\begin{array}{cc} 1 & \text{if}\;\;\left\{p_{i}\in g_{j}\right\}\\ 0 & \text{if}\;\;\left\{p_{i}\notin g_{j}\right\} \end{array}\right. \end{array}$$

$$\begin{array}{cc} \left(\text{b}\right) & C_{ij}=\left\{\begin{array}{cc} \frac{1}{k} & \text{if}\;\;\left\{p_{i}\in g_{j}\right\}\\ 0 & \text{if}\;\;\left\{p_{i}\notin g_{j}\right\}, \end{array}\right. \end{array}$$

where *p*_*i*_ is the *i*th probe and *g*_*j*_ represents the extended *j*th gene region.

**Step 5 (generate CNAR datasets).**

We combined all CNA datasets from Step 1 into a single large matrix with probes in rows and samples in columns. Next, we produced new CNAR datasets as a simple product of matrices via multiplication of one of the base matrices from Step 4 and a CNA data matrix. Finally, we generated new CNAR datasets in matrix form, presenting CNV in terms of gene names.

**Figure 1:**
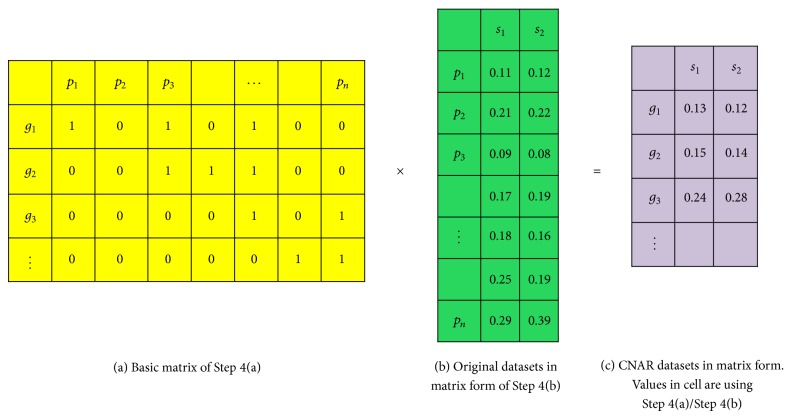
Procedure for generation of CNAR datasets. (a) Basic matrix in Step 4, (b) original datasets of copy number alteration for each probe. By multiplying (a) and (b), CNAR datasets were generated.

These comprehensive datasets allow a large class of gene-expression software to be utilized to study DNA rather than RNA signatures. In Figure 1, we illustrate how this software works and demonstrate its application.

## 3. Results

To test our newly generated CNAR datasets, we implemented well-known machine learning algorithms including unsupervised cluster methods and supervised classification methods: consensus clustering, silhouette clustering, and the support vector machine (SVM). The Fisher criterion method [13] was adopted for ranking subsets of genes to be evaluated: (*μ*_*A*_ − *μ*_*B*_)^2^/(*σ*_*A*_^2^ + *σ*_*B*_^2^), representing the square of the difference in means of two classes (*A*, *B*) divided by the sum of the square of their variances.

### 3.1. Application to Classification Methods (SVM) and Feature Selection

We applied the newly generated CNAR datasets to SVM with feature selection for classification of survival in patients with OV (18 short and 18 long samples) and GBM (23 short and 23 long samples). To avoid any prior bias, the training of the classifier, the choice of the number of features, and feature selection were done strictly in test datasets. For evaluation, we used standard leave-one-out cross validation (LOOCV). In OV, the best classification accuracy of long versus short survival was 83.33%, using four features. For GBM, the best classification accuracy of long versus short survival was 82.61%, using 20 selected features. The accuracies obtained using CNAR datasets were higher than the accuracies obtained using gene-expression microarray datasets (63.64% and 72.35% for OV and GBM, resp.).

**Figure 2:**
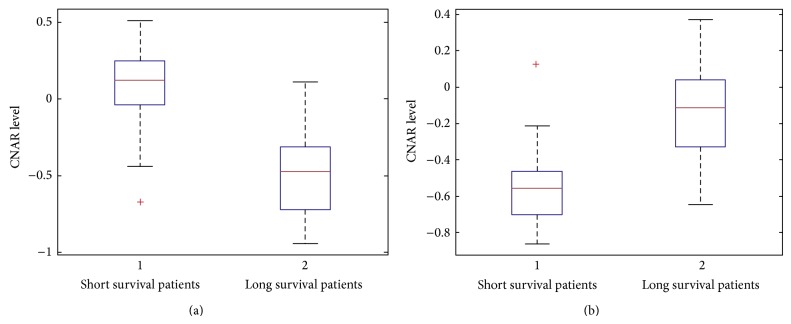
(a) Plot for STOML3 in short and long classes of OV patients using CNAR datasets. (b) Plot for ZNF488 in short and long classes of GBM patients. The *y*-axis represents CNAR level; the *x*-axis represents patients classes 1: short, 2: long.

In OV datasets, since four-feature selection performed best results, we collected all selected four features from all train datasets as performing LOOCV. STOML3, including eight CN probes from chr13:39482884 to chr13:39554832, was selected in all training samples. The boxplots of STOML3 show the comparison of short survival patients to long survival patients (Figure 2(a)). In general, genes with increases in copy number represent oncogenes, and genes with decreases in copy number represent tumor suppressors, which affect short and long survival time, respectively. As shown in the figure, mutation of this gene may cause cancer: if copy number increases, then survival time is short, whereas if the gene is lost, survival time is long. We also selected the most significant gene, ZNF488, from the short and long class GBM CNAR datasets. Our pseudogene region of ZNF488 is from chr10:48355024 to chr10:48373866, including only one probe.  Figure 2(b) shows the comparison of short and long survival patients. Since ZNF488 is related with antibody, the boxplot shows consistency of the mechanism.

### 3.2. Application to Clustering Methods (Consensus and Silhouette Clustering)

**Figure 3:**
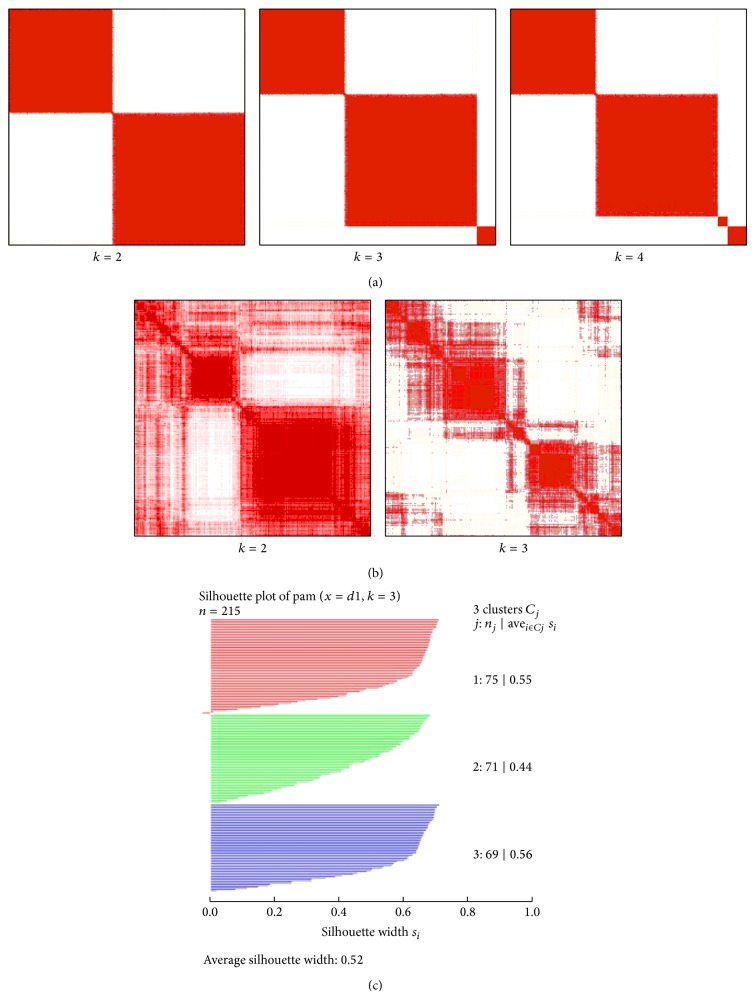
(a) Consensus clustering using CNAR datasets with 204 genes from 215 GBM samples from *k* = 2 to 4. (b) Consensus clustering using CNAR datasets with 1604 genes from 1740 genes selected from Verhaak et al. [11]. (c) Silhouette clustering plot using 1604 out of 1740 genes from CNAR.

We applied the CNAR datasets to the unsupervised consensus clustering algorithm [14] provided in GenePattern [15] and Silhouettes [16]. We tested 215 GBM samples with 204 genes out of 21,856 pseudogenes, setting an arbitrary cutoff of the standard variance at 0.4.  Figure 3 shows GBM clustering using CNAR datasets. Consensus clustering in GenePattern using CNAR datasets of the 204 selected GBM genes is shown in Figure 3(a). We extracted 1740 genes in Verhaak et al. [11] which used mRNA expression GBM datasets downloaded from TCGA. We also extracted CNAR datasets of the 1740 genes and applied the datasets to consensus clustering shown in Figure 3(b). In addition, we demonstrated the performance of silhouette clustering in Figure 3(c). We compared the clustering result of CNAR datasets to the clustering result of mRNA expression datasets in Verhaak et al. [11] using the same 1740 genes. The average silhouette width is 0.18 in Verhaak et al., and our CNAR datasets yielded a silhouette width of 0.52. Thus, CNAR datasets yielded a superior clustering performance relative to microarray expression data.

**Figure 4:**
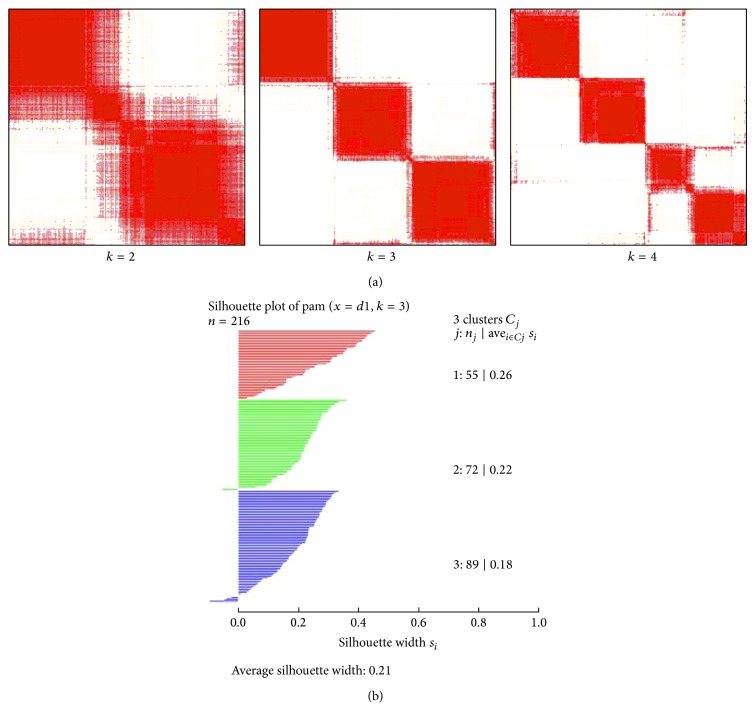
(a) Consensus clustering using CNAR datasets with 175 genes from 216 OV samples from *k* = 2 to *k* = 4. (b) Silhouette clustering plot using the same (a) datasets.

We also subjected OV CNAR datasets to the same procedure. In that case, we downloaded 216 samples and selected 175 genes with variance of 0.4, and the results are shown in Figure 4 with a silhouette width of 0.21. Even though clustering methods clearly separated the samples into three groups, those groups were not related to survival time: specifically, Kaplan-Meier survival curve analysis of these three clustering groups did not reveal significant differences (*P* = 0.09; data not shown).

**Figure 5:**
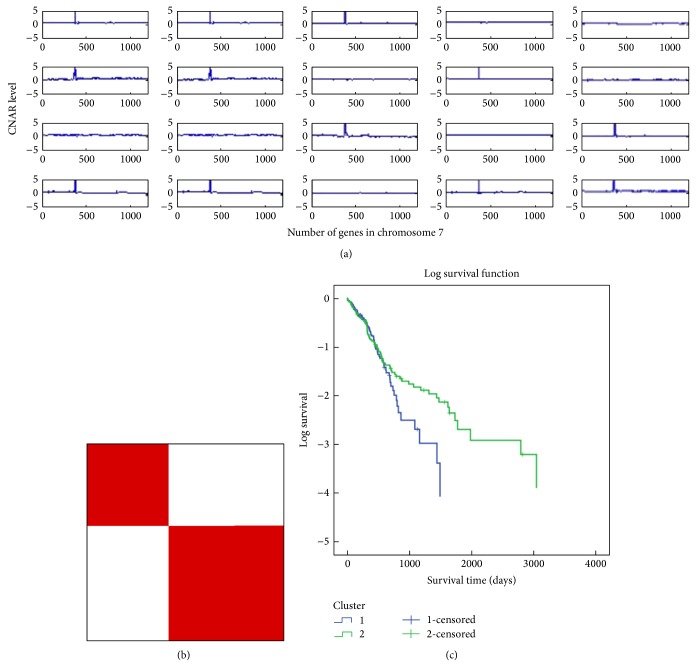
(a) Examples of CNAR across chromosome 7 for selected patients. The *y*-axis denotes the CNAR levels, and the *x*-axis denotes the number of pseudogenes in chromosome 7. (b) Consensus clustering of EGFR CNAR datasets. (c) Kaplan-Meier survival analysis of EGFR clusters given by (b) (log-rank *P* value = 0.28).

In addition, we selected the gene with the largest gain in copy number, EGFR, using standard deviation from the GBM CNAR dataset. The distribution of CNAR in chromosome 7 is provided in Figure 5(a) for randomly selected 20 out of 215 samples. The *y*-axis represents CNAR level; the *x*-axis represents genes in chromosome 7. Figure 5(a) reveals two distinct classes plotting EGFR which includes 38 probes in the regions chr7:54990071–chr7:55414419 in chromosome 7. We applied the gene EGFR CNAR dataset to the consensus clustering method provided in GenePattern [15] shown in Figure 5(b). The plot is clearly distinct in two classes. Kaplan-Meier plot of two distinct classes given by Figure 5(b) is shown in Figure 5(c).

### 3.3. Application to Gene Set Enrichment Analysis

We subjected CNAR datasets derived from OV and GBM samples to gene set enrichment analysis (GSEA) [17]. The OV CNAR survival datasets included 18 short class and 18 long class samples, and the GBM datasets included 23 short class and 23 long class samples.  Table 1 shows the most enriched pathways in OV and GBM, with a FDR cutoff of zero. The most enriched pathways in both cancers were related to metabolism. In particular, the pentose and glucuronate interconversion pathway and porphyrin and chlorophyll metabolism were enriched in both OV and GBM.

## 4. Discussion 

In this study, we generated new datasets that represented DNA variation in formats that paralleled variation in RNA expression and subjected the data to established machine learning algorithms. Most previous studies identified changes in DNA using CN alteration and then interpreted the relationship with genes by looking up the chromosome region. These new CN alteration-based datasets, which we call CN array (CNAR) datasets, could be used directly for identification of genetic variation at the RNA level. In addition, the new CNAR datasets enable straightforward visualization of gain or loss of pseudogenes along the chromosome. In our analyses, we used several existing methods including clustering, classification, and GSEA. In addition, we applied CNAR datasets to two clustering methods, consensus clustering and silhouette clustering. The clustering performances were superior to those obtained using RNA expression. We also applied CNAR to the SVM classification algorithm for GBM and OV cancer patients stratified for short and long survival times; the accuracies were 83.33% and 82.61%, respectively. When we subjected CNAR datasets to GSEA, we found two enriched metabolic pathways in both OV and GBM with a FDR cutoff of zero. These new datasets enable many applications, including clustering of cancer subtypes, prediction of survival times, and classification of cancer metastasis by analyzing DNA alterations using tools developed for RNA-level analysis. Such analyses may provide novel insights into the biological mechanisms underlying cancer. The major limitation of this analysis is the fixed extension of the gene region. Up to date there is no information for extending gene region for copy number alteration. Therefore, we need further study for extension method for each gene region.

## Figures and Tables

**Table 1:** Pathways enriched in OV and GBM: categories of molecular interaction and reactions, from KEGG, are indicated: (1.1) carbohydrate metabolism, (1.2) energy metabolism, (1.3) lipid metabolism, (1.8) metabolism of cofactors and vitamins, (1.9) metabolism of terpenoids and polyketides, (1.11) xenobiotic biodegradation and metabolism, and (2.2) translation.

Pathways enriched in OV	FDR	Enriched in class
Pentose and glucuronate interconversion (1.1)	0	Long
Androgen and estrogen metabolism	0	Long
Porphyrin and chlorophyll metabolism (1.8)	0	Long
Aminoacyl tRNA biosynthesis (2.2)	0	Long
Carbon fixation in photosynthetic organisms (1.2)	0	Long
3-Chloroacrylic acid degradation	0	Short
Caprolactam degradation (1.11)	0	Short
Glycolysis and gluconeogenesis (1.1)	0	Short
Atrazine degradation (1.11)	0	Short
Polycyclic aromatic hydrocarbon degradation (1.11)	0	Short

Pathways enriched in GBM	FDR	Enriched in class

Linoleic acid metabolism (1.3)	0	Short
Arachidonic acid metabolism (1.3)	0	Short
Terpenoid backbone biosynthesis (1.9)	0	Short
Ether lipid metabolism (1.3)	0	Short
Pentose and glucuronate interconversion (1.1)	0	Long
Porphyrin and chlorophyll metabolism (1.8)	0	Long
